# Reduced Dose Perioperative Non‐Steroidal Anti‐Inflammatory Drugs in Arthroplasty Patients With Renal Impairment: A Five‐Year Cohort Study

**DOI:** 10.1111/ans.70261

**Published:** 2025-07-25

**Authors:** Liam T. A. Fernando‐Canavan, Alistair Stanny, John Richmond, David J. Mitchell

**Affiliations:** ^1^ Grampians Health Ballarat Orthopaedic Unit Ballarat Victoria Australia; ^2^ Health Economics and Simulation Modelling for Chronic Disease Unit, Melbourne School of Population and Global Health University of Melbourne Melbourne Australia; ^3^ Ballarat Clinical School, School of Medicine Deakin University Ballarat Australia; ^4^ Novar Musculoskeletal Research Institute, Ballarat Orthopaedic & Sports Medicine Ballarat Victoria Australia

**Keywords:** acute kidney injury, arthroplasty, multimodal analgesia, non‐steroidal anti‐inflammatory drugs, perioperative care

## Abstract

**Background:**

Non‐steroidal anti‐inflammatory drugs (NSAIDs) are fundamental for multimodal analgesic therapy, but are traditionally avoided in renally impaired patients. We aim to show NSAIDs can be safely administered to arthroplasty patients with a pre‐operative estimated glomerular filtration rate (eGFR) less than 60 mL/min/1.73 m^2^ without causing a permanent reduction in post‐operative eGFR and, in doing so, minimise opioid use.

**Methods:**

We prospectively collected data from patients with a pre‐operative eGFR less than 60 mL/min/1.73 m^2^ undergoing arthroplasty between January 2018 and June 2023 at St John of God Ballarat Hospital, Australia. Baseline eGFR was obtained pre‐operatively, and on at least 1 of days 1, 2, 10, or a follow‐up review appointment of at least 4 weeks. Patients received tailored dosing of perioperative NSAIDs corresponding to pre‐operative eGFR. Acute kidney injury (AKI) was defined using the RIFLE criteria.

**Results:**

We identified 221 patients. Median baseline eGFR was 50 mL/min/1.73 m^2^ and median eGFR recovery at latest follow‐up was 109% of baseline eGFR. 28% of the cohort had a clinically significant reduction in eGFR on day 1 post‐operatively, and by latest follow‐up, this subgroup had a median recovery of 106% of baseline eGFR. At the 2‐week follow‐up, there were no cases of AKI, and by latest follow‐up, no patients had a clinically significant reduction of baseline eGFR.

**Conclusion:**

Perioperative NSAIDs in renally impaired patients undergoing arthroplasty surgery can be safely given using an adjusted dose protocol based on pre‐operative renal function. Future studies investigating the circumspect use of NSAIDs for pain management during arthroplasty are warranted.

## Graphical abstract

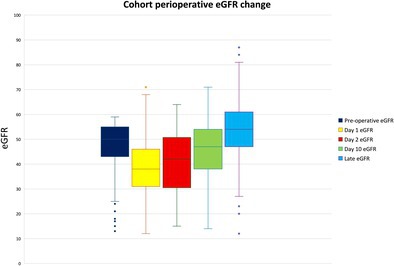

## 1 Introduction

A key element of the rapid recovery and rehabilitation principles of short stay arthroplasty is multimodal analgesia [1, 2], and it has been demonstrated that following arthroplasty, the use of post‐operative non‐steroidal anti‐inflammatory drugs (NSAIDs) not only ensures earlier discharge, but also a faster accomplishment of range of motion and ambulatory goals, less reliance on opioids, and reduced post‐operative complications [3, 4, 5].

Whilst NSAIDs are known to be more cost‐effective than opioids for treating osteoarthritis [6], their use is traditionally avoided in the acute phase of treatment for patients with impaired renal function undergoing arthroplasty due to concerns of an increased risk of acute kidney injury (AKI) [7, 8, 9]. However, a recent Cochrane review showed NSAIDs have uncertain effects on the risk of post‐operative AKI [10], and NSAIDs such as ketorolac have been demonstrated to have no significant effects on renal function during the post‐operative period [11, 12, 13, 14].

To our knowledge, there is no published research investigating the circumspect use of NSAIDs in patients with pre‐existing renal impairment undergoing arthroplasty. In this study we therefore asked: (1) can NSAIDs be safely administered to arthroplasty patients with a pre‐operative eGFR less than 60 mL/min/1.73 m^2^ and in doing so, (2) have no permanent reduction on post‐operative eGFR and (3) minimise the use of opioids.

## 2 Methods

### 2.1 Study Population

An analysis of prospectively collected eGFR results was conducted using hospital data, which was inclusive of pathology laboratory results, medication and anaesthetic charts, and pharmacy records for patients with pre‐existing renal impairment undergoing all primary or revision hip, knee, and shoulder arthroplasty between January 2018 and June 2023 at a single institution (St John of God Ballarat Hospital, Australia). All procedures were performed by a single surgeon.

Patients were hydrated pre‐operatively with a 600 mL isotonic carbohydrate‐electrolyte solution up until 2 h prior to surgery, a minimum of 1 L intravenous fluids intraoperatively, and immediate oral rehydration post‐operatively, beginning in the post‐anaesthetic recovery bay. We did not evaluate any perioperative reductions in urine output. Patients using angiotensin‐converting enzyme inhibitors (ACEi) or angiotensin II receptor blockers (ARBs) had their medication withheld on the day of surgery and during their admission to hospital if blood pressure remained less than 130/80 mmHg.

### 2.2 Approach to Perioperative Medication Management

Local infiltration analgesia (LIA) described by Kerr & Kohan was used routinely; ropivacaine 300 mg in 150 mL, ketorolac 30 mg, adrenaline 0.5 mg, 1 g tranexamic acid, and 4 mg dexamethasone [15]. Perioperative NSAIDs were prescribed based on pre‐operative eGFR using a standardised protocol (Table 1) developed by the senior author (D.J.M.) and a nephrologist (co‐author, J.R.). This protocol was unchanged per procedure. All patients received an intraoperative periarticular dose of ketorolac 30 mg and a 0.2 μm filtered intra‐articular wound catheter to allow for post‐operative intra‐articular ketorolac 30 mg per protocol (Table 1), dexamethasone 4 mg, and ropivacaine 100 mg. Tranexamic acid was used to minimise blood loss by using 1 g oral pre‐operatively, 1 g in the LIA periarticular mix, and topically applied 1 g intra‐articular at the conclusion of surgery. Where knee arthroplasty was undertaken, no tourniquet was used.

**TABLE 1:** Perioperative NSAID dosing protocol.

Pre‐operative eGFR (mL/min/1.73 m^2^)	Intraoperative dose	Evening dose	Morning dose	Discharge dose
> 60	Ketorolac 30 mg	Ketorolac 30 mg^a^	Ketorolac 30 mg	Meloxicam 7.5 mg twice daily^b^
30–59	Ketorolac 30 mg	—	Ketorolac 30 mg	Meloxicam 7.5 mg daily^b^
< 30	Ketorolac 30 mg	—	—	—
Renal replacement therapy	Ketorolac 30 mg	—	—	Meloxicam 7.5 mg daily^b^

^a^ If operated on a morning theatre list.

^b^ If patients already used an alternative regular NSAID to meloxicam 7.5 daily, they would be discharged with this usual dose of NSAID.

Oral meloxicam 7.5 mg per protocol (Table 1) was prescribed on drug charts throughout admission and continued on discharge. If patients used an alternative regular NSAID to meloxicam 7.5 mg (e.g., celecoxib 100 mg), they were instead discharged with their usual NSAID. If there was a known baseline eGFR of less than 30 mL/min/1.73 m^2^, patients would not be discharged with an oral NSAID. Buprenorphine 5 μg/h was used for total knee arthroplasty and posterior approach total hip arthroplasties; the first patch was applied for 6 days, followed by a second patch for 10 days, which would then be removed. If a patient was using regular oral opioids prior to surgery purely for the joint being replaced, the dose was reduced by 50% from the date of surgery, and a further 50% reduction a week later, then stopped. If a patient was on a regular oral opioid for pain unrelated to osteoarthritis, this dose was unchanged. Oral opioids were only included on drug charts as a PRN dose to be used in circumstances of severe pain unresolved by NSAIDs. Oxycodone was avoided, preferring tramadol or tapentadol for patient‐initiated top‐up (PRN) analgesia. Patients were routinely prescribed pantoprazole 20 mg daily, unless already on a larger dose or equivalent proton‐pump inhibitor. Patients were routinely prescribed Macrogol 3350 sachets twice daily.

### 2.3 Inclusion Criteria

Patients were included in the study if they had a pre‐operative eGFR less than 60 mL/min/1.73 m^2^ and were undergoing hip, knee, or shoulder joint arthroplasty surgery from January 2018 to June 2023 at St John of God Ballarat Hospital, Australia.

### 2.4 Exclusion Criteria

Patients with a pre‐operative eGFR greater than 60 mL/min/1.73 m^2^ or those undergoing renal replacement therapy prior to surgery were excluded from the study. No other patients (e.g., those on diuretics or ACEi/ARBs) were excluded from the study.

### 2.5 Data Collection

Patient eGFR results were used as a marker for renal function using the RIFLE (Risk of renal dysfunction; Injury to the kidney; Failure of kidney function, Loss of kidney function and End‐stage kidney disease) criteria, where a greater than 25% decrease of baseline eGFR is considered at risk of AKI, and greater than 50% is considered an AKI [16].

All patients had a baseline eGFR measurement during a pre‐operative appointment between 1 week and 3 months before surgery. No pre‐operative changes were made to regular NSAID usage following this baseline eGFR measurement. Post‐operative eGFR was measured on at least 1 of days 1, 2, 10, or a follow‐up review appointment of at least 4 weeks. Any drop in eGFR of greater than 25% of the baseline renal function on post‐operatively was deemed to be a clinically significant reduction in eGFR as per the RIFLE criteria and greater than 50% of baseline eGFR was considered an AKI [16]. Any patient who did not return to at least 75% of their baseline eGFR (i.e., no decrease of greater than 25% of baseline eGFR) on Day 1 post‐operatively underwent further eGFR measures on Day 2 and again on day 10 before their 2 week post‐operative review appointment if the Day 2 result was not above 75% of baseline eGFR. If the day 10 eGFR result had still not returned to pre‐operative levels, a further eGFR measurement was taken at a follow‐up review appointment of at least 4 weeks post‐operatively (‘late eGFR’). Any patient who had returned to within 75% of baseline eGFR post‐operatively and was therefore no longer at risk of AKI, did not undergo further eGFR testing on sequential post‐operative days, and so we did not have serial eGFR measures for all patients [17].

Further data was collected for the whole cohort inclusive of date of birth, gender, weight, length of stay, type of arthroplasty (shoulder, hip, knee) and whether the procedure was performed as a primary or revision arthroplasty. Patients having knee arthroplasty were also recorded as being either total or unicompartmental procedures, and patients who underwent multiple arthroplasty procedures at different time periods (e.g., both a THA and a TKA) were included as individual inpatient separations. We also reviewed intraoperative anaesthetic charts and inpatient medication charts to collect data on the full perioperative course of medication dosing pertaining to NSAIDs. This study adheres to the STROBE guidelines for reporting observational studies [18] (Appendix 1).

### 2.6 Statistical Analysis

The primary study outcome was the effect of NSAIDs on eGFR. Following arthroplasty, baseline eGFR was compared to an eGFR measurement taken on 1 or more of days 1, 2, 10, or at least 4 weeks postoperatively. Results were presented in terms of actual eGFR and percentage change in eGFR as compared to baseline eGFR. Further subgroup analysis was undertaken on individuals who had a reduction of 25% or more of baseline eGFR following arthroplasty, corresponding to the RIFLE criteria.

## 3 Results

During the study time period, 221 patients undergoing arthroplasty were found to have a pre‐existing eGFR of less than 60 mL/min/1.73 m^2^ and included in our study (Figure S1). 58% of patients were female, and the mean age of our cohort was 78 years (Table S1). Of the cohort of 221 patients, 126 were treated with TKA, 87 by THA, and 8 were shoulder arthroplasties. 91% of cases included were primary arthroplasties, 12 of whom had a more than 1 primary arthroplasty (e.g., one patient undergoing THA and TKA at two different time points). Twenty cases were revision arthroplasty (9%). The median baseline eGFR for our cohort was 50 mL/min/1.73 m^2^, with 69% of the patient cohort having an eGFR between 45 and 59 mL/min/1.73 m^2^, corresponding to Grade 3a CKD (Table S1).

Our protocol (Table 1) was designed for all patients to receive intraoperative periarticular ketorolac 30 mg; however, 19% of patients received additional NSAIDs than our protocol dosing as they were administered IV parecoxib 40 mg per review of patient anaesthetic charts. 88% of patients were given a day 1 post‐operative periarticular ketorolac 30 mg dose in the morning after their procedure. 95% of the cohort had a regular oral NSAID charted during their admission, and 88% were discharged with a regular oral NSAID. For 95% of the inpatients charted a regular oral NSAID, the majority were given meloxicam 7.5 mg daily (75%), with meloxicam 7.5 mg daily also being the most common discharge NSAID (57%), followed by meloxicam 7.5 mg twice daily (25%). Four patients with an eGFR less than 30 mL/min/1.73 m^2^ were noted to have been charted regular meloxicam 7.5 mg, and three of these patients were also discharged with meloxicam 7.5 mg; all had returned to greater than 100% of their baseline eGFR by the latest follow‐up.

21% of patients had an opioid intolerance, most commonly codeine (10%), morphine (6.8%) tramadol (7.7%), or oxycodone (5%). For the duration of inpatient stay, 88% of all patients were prescribed a PRN dose of tramadol 50 mg immediate‐release, with the next most commonly prescribed PRN analgesic being tapentadol 50 mg immediate‐release (8%). Oxycodone as a 5 mg or 10 mg preparation, or in combination with naloxone, was prescribed in 6% of patients. The median dose used of oral PRN analgesic was one tablet, while 38% of patients did not use any oral PRN analgesic. In the cohort of patients with an eGFR < 30 day one post‐operatively (*n* = 42), their median dose of oral PRN analgesic was two tablets, and most commonly this was tramadol 50 mg immediate‐release (17% of patients). 41% of the patients with an eGFR < 30 day one post‐operatively did not use any PRN analgesic. Of the 20 cases of revision arthroplasty, this cohort used a median of one and a half PRN oral analgesics, again most commonly tramadol 50 mg immediate‐release (60%). 30% of revision cases did not use any oral PRN analgesics.

**FIGURE 1:**
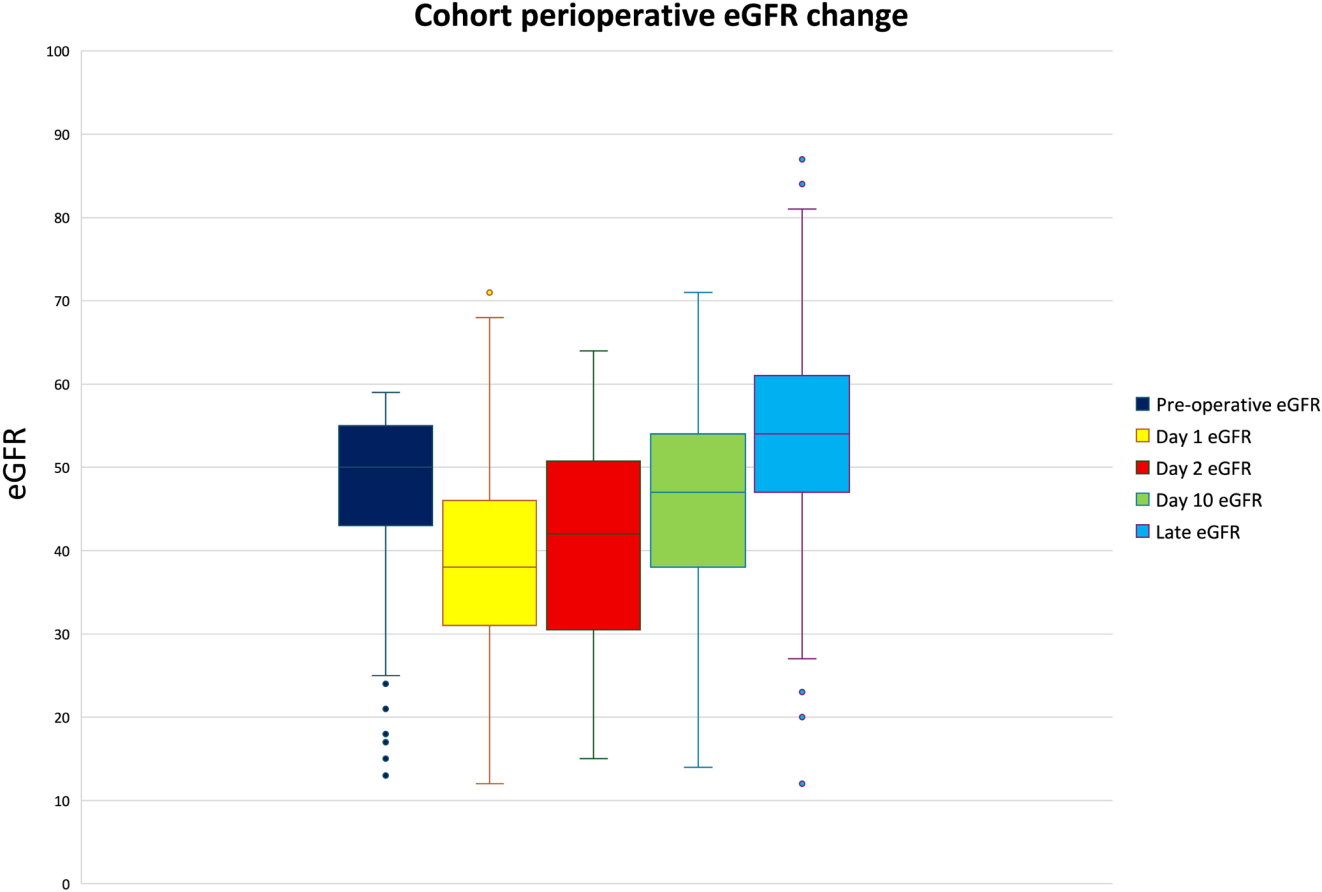
Change in median eGFR from baseline; eGFR measured as mL/min/1.73 m^2^.

Figure 1 shows all patients with available eGFR results and their corresponding eGFR changes from baseline at each timepoint. As mentioned above, the median baseline eGFR measured pre‐operatively for our cohort was 50 mL/min/1.73 m^2^ (*n* = 221). The median post‐operative eGFR on Day 1 fell to 38 mL/min/1.73 m^2^ (*n* = 176) and then increased to a median 47 mL/min/1.73 m^2^ at the 2‐week post‐operative appointment (*n* = 150), and a median 54 mL/min/1.73 m^2^ at the late eGFR measurement (*n* = 89). To better understand the cohort change in eGFR, we investigated the percentage of recovery to baseline eGFR in Figure S2. On Day 1 post‐operatively, the interquartile range of eGFR recovery for our cohort was between 70% and 95%, meaning half of the cohort had recovered to between 70% and 95% of baseline eGFR. At the 2‐week post‐operative review (i.e., eGFR measured day 10 post‐operatively), the cohort median recovery to baseline eGFR was 100%, with an interquartile range between 111% and 91% of baseline eGFR. The interquartile range for late eGFR measurement in Figure S2 showed a marginal increase in recovery to between 119% and 98%, while the median eGFR recovery at late eGFR for the cohort was 109%.

**FIGURE 2:**
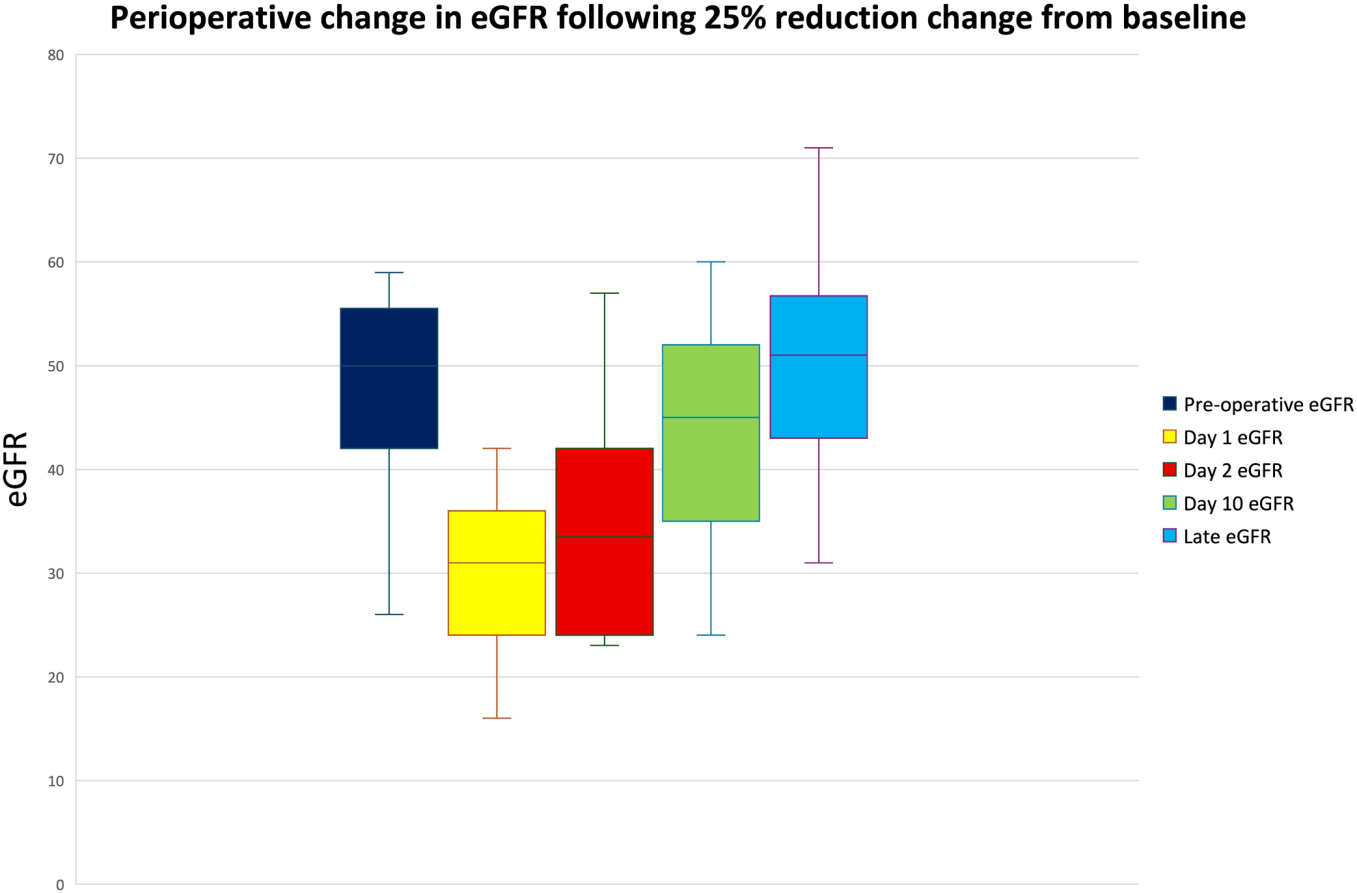
Change in median eGFR following 25% baseline eGFR reduction; eGFR measured as mL/min/1.73 m.

We conducted further analysis into the 28% (*n* = 61) of our cohort who had a reduction of more than 25% of their baseline eGFR on Day 1 post‐operatively. Figure 2 shows the median baseline eGFR for this subgroup was 50 mL/min/1.73 m^2^ (*n* = 61), and their median post‐operative eGFR fell to 31 mL/min/1.73 m^2^ on day 1 post‐operatively (*n* = 61). The eGFR for this subgroup then increased to a median of 45 mL/min/1.73 m^2^ at the 2‐week post‐operative appointment (*n* = 51), followed by a median of 51 mL/min/1.73 m^2^ at the late eGFR measurement (*n* = 28). On Day 1, for the percentage recovery to baseline eGFR for this subgroup (Figure S3), the interquartile range was between 60% and 70%, and by the 2‐week post‐operative review, the interquartile range rose to between 86% and 107%. The median eGFR recovery at the late eGFR measurement for this subgroup was 106%. All patients within this subgroup had recovered to above 75% of eGFR by the latest measure.

Four patients had a greater than 50% reduction of their baseline eGFR on day 1 (52%, 53%, 56%, and 57%), corresponding to AKI using the RIFLE criteria. All four had a pre‐operative eGFR greater than 30 mL/min/1.73 m^2^, were aged above 75 years and all underwent primary arthroplasty. By the two‐week post‐operative review, all 4 patients had recovered to above 75% of baseline eGFR.

## 4 Discussion

We sought to demonstrate circumspect use of NSAIDs on a cohort of patients with known renal impairment can be safely administered. As seen in Figure 1, while there was a transient decrease in eGFR Day 1 post‐operatively for our cohort, this was followed by an observable increase in eGFR with the late eGFR measurement being 111% of baseline eGFR. For the subgroup of 28% of our cohort who had a reduction of more than 25% of baseline eGFR (‘at risk’ of AKI using the RIFLE criteria), on day 1 post‐operatively, the median eGFR recovery by the late eGFR measurement was 106% of baseline eGFR. No patients within this subgroup had a greater than 25% reduction of baseline eGFR by the latest eGFR measure.

There were four out of 221 patients who had a greater than 50% reduction of baseline eGFR on Day 1, corresponding to an AKI using the RIFLE criteria. Further to this, if there was indeed an intrinsic renal AKI, we would have expected a progressive worsening of eGFR on Days 2 and 10 given the delayed response in achieving steady‐state concentration of serum creatinine seen with the renal kinetics of CKD patients [19]. However, as prostaglandin production is increased in patients with CKD to improve the perfusion of their remaining nephrons, these reductions in eGFR are highly likely due to the reversible effects of NSAIDs inhibiting COX‐2 and therefore production of the prostaglandins which would otherwise vasodilate the afferent arteriole of the glomerulus to protect intraglomerular blood flow [20]. Given all four of these patients went on to recover above 75% of their baseline eGFR (i.e., no longer ‘at risk of AKI’ using the RIFLE criteria) by the 2‐week post‐operative review without any specific treatment for an AKI, it is unlikely that these four patients actually sustained an irreversible ischaemic tubular injury which could worsen their pre‐existing CKD. It is therefore likely that these four patients did not suffer intrinsic damage to the kidney, rather, they had a transient functional impairment relative to their physiologic demands, contrary to the definition of AKI as a persistent injury and corresponding to a ‘transient AKI’ using more modern definitions of acute kidney disease and renal recovery [21]. Our findings therefore suggest the circumspect use of NSAIDs in a cohort of patients undergoing arthroplasty was safely administered. Further to this, our results may underestimate the true cohort recovery in eGFR given we did not conduct serial eGFR measurements in those who had already regained their renal function post‐operatively.

38% of all patients and 41% of patients with a Day 1 post‐operative eGFR < 30 did not use any PRN oral analgesics while an inpatient. Of those who did use a PRN oral analgesic, this was most commonly tramadol 50 mg immediate‐release (88% of all patients), and a median of one tablet overall was used throughout the entire admission. For the cohort with a reduced eGFR < 30 post‐operatively on day 1, a median of two tablets overall was used throughout the entire admission. Results were similar for the revision arthroplasty cohort, with a median of one and a half PRN oral analgesics used and 30% of patients using no PRN analgesics at all. Oxycodone as a 5 or 10 mg preparation or in combination with naloxone was avoided and overall it was used in 6% of patients. In a cohort of post‐operative arthroplasty patients with reduced eGFR who would traditionally make use of opioids for management of acute post‐operative pain, our analgesic protocol which emphasises the use of NSAIDs suggests an intended consequence of reduced reliance on opioids for post‐operative analgesia.

There are several limitations to be considered. Inpatients were continued on their regular pre‐operative NSAIDs and therefore the dose and frequency of meloxicam and celecoxib as inpatients was not standardised, while outpatient NSAID use was not able to be accurately measured. Pain scores (e.g., a visual analogue scale) were not included as an objective measure of pain or to determine the need for PRN analgesia. As this study was performed at a single hospital and all patients were operated on by a single surgeon, our findings may not be applicable to other centres in different geographical areas. Further to this, the sample size (*n* = 221) and lack of a case–control population for comparison may undermine the internal and external validity of our findings. As we did not complete serial eGFR measurements for the entire cohort, we were not able to determine if recovery in baseline eGFR was statistically significant across each of the measurements (e.g., Day 1 eGFR versus Day 10 eGFR). Lastly, AKI occurring after discharge and discovered in the community may have been treated in the community, or as a second episode of hospital care, which was not captured in the present study and would be an unlikely scenario based on the data that has been analysed.

While this study supports our proposition that it is safe to give NSAIDs during the perioperative management of arthroplasty in patients with renal impairment using a tailored regimen corresponding to pre‐operative renal function, this is a small cohort study conducted at a single institution. Further to this, as our study primarily included patients using doses of ketorolac and meloxicam, given that the side effects of NSAIDs may vary with different preparations, generalisations cannot be made from this single study. We would therefore advocate for further investigation of the impact of NSAIDs on arthroplasty outcomes. Future research should also consider the use of measurement of pain scores, fluid balance such as fluid input and output, or intra‐operative blood loss, costs, and health‐related quality of life.

## 5 Conclusion

Our findings suggest that the perioperative use of NSAIDs in patients with known renal impairment undergoing arthroplasty surgery can be safely given using an adjusted dose protocol based on pre‐operative renal function. Care and consideration must be taken to appropriately adjust dosing and monitor for clinically significant reduction in eGFR during the early post‐operative period. Future studies investigating the circumspect use of NSAIDs for pain management during arthroplasty are warranted.

## Author Contributions

**Liam T. A. Fernando‐Canavan:** data curation, formal analysis, methodology, writing – original draft, writing – review and editing. **Alistair Stanny:** data curation, investigation, writing – original draft. **John Richmond:** conceptualization, methodology, supervision, writing – review and editing. **David J. Mitchell:** conceptualization, investigation, methodology, project administration, supervision, writing – original draft, writing – review and editing.

## Ethics Statement

This study received ethics approval from the Ballarat Health Services and St John of God Healthcare Human Research Ethics Committee (HREC no. LNR/62248/BHSSJOG‐2020‐215 869).

## Conflicts of Interest

The authors declare no conflicts of interest.

## Supporting information


**Appendix S1.**Supporting Information.

**Figure S1.** Patient flow chart.

**Figure S2.** Percentage change in median post‐operative eGFR as compared to baseline eGFR.

**Figure S3.** Percentage change in median eGFR following 25% reduction from baseline eGFR.

**Table S1.** Cohort demographics.
