# Supplementary figures and images for: Reduced Dose Perioperative Non‐Steroidal Anti‐Inflammatory Drugs in Arthroplasty Patients With Renal Impairment: A Five‐Year Cohort Study
Source: ANZ J Surg. 2025 Jul 25;95(7-8):1553–9. doi: 10.1111/ans.70261 (PMC12413587; doi:10.1111/ans.70261)

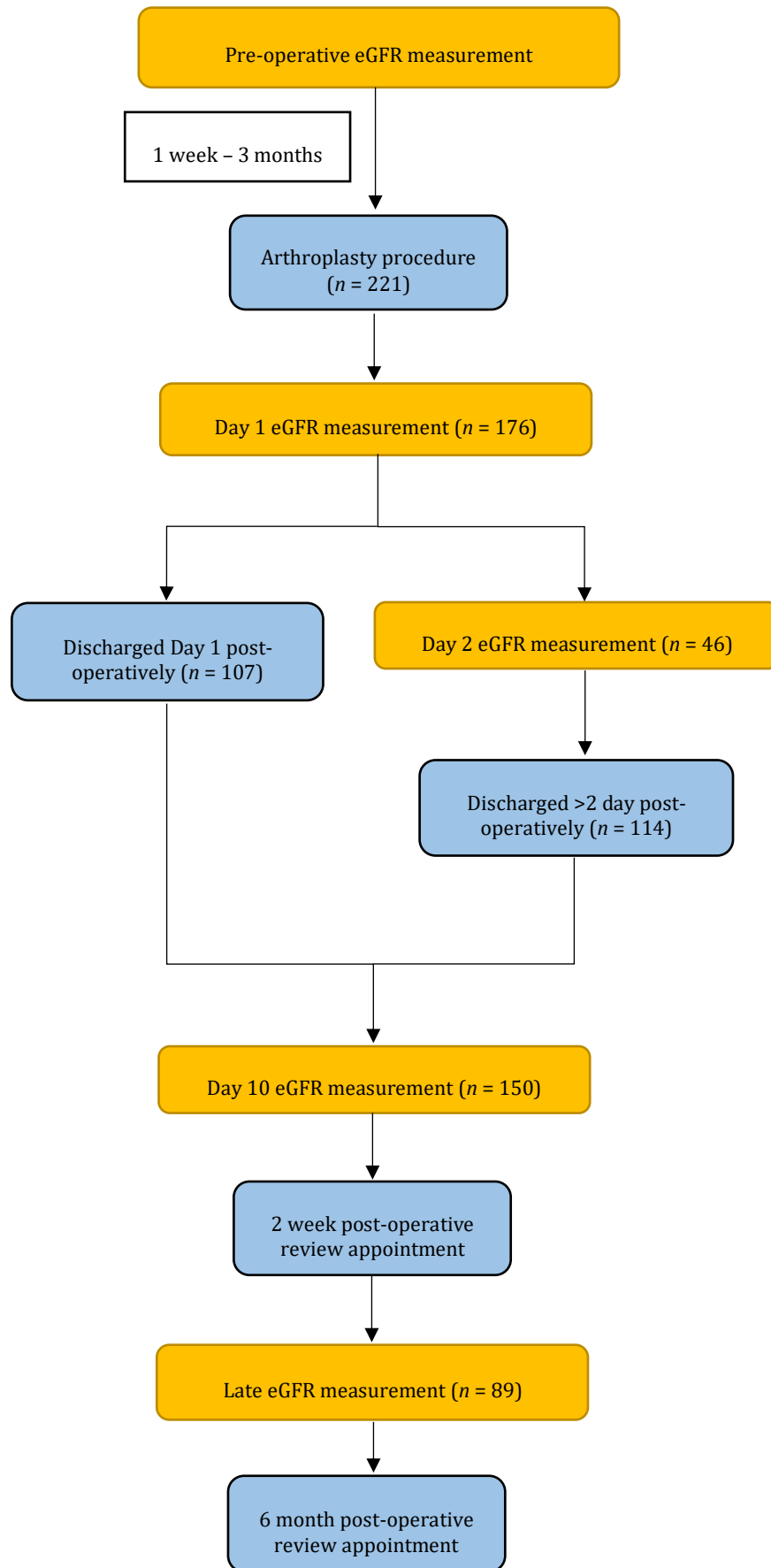

Supplement: Supplementary file 2 — Figure S1. Patient flow chart. [file ANS-95-1553-s001.pdf]

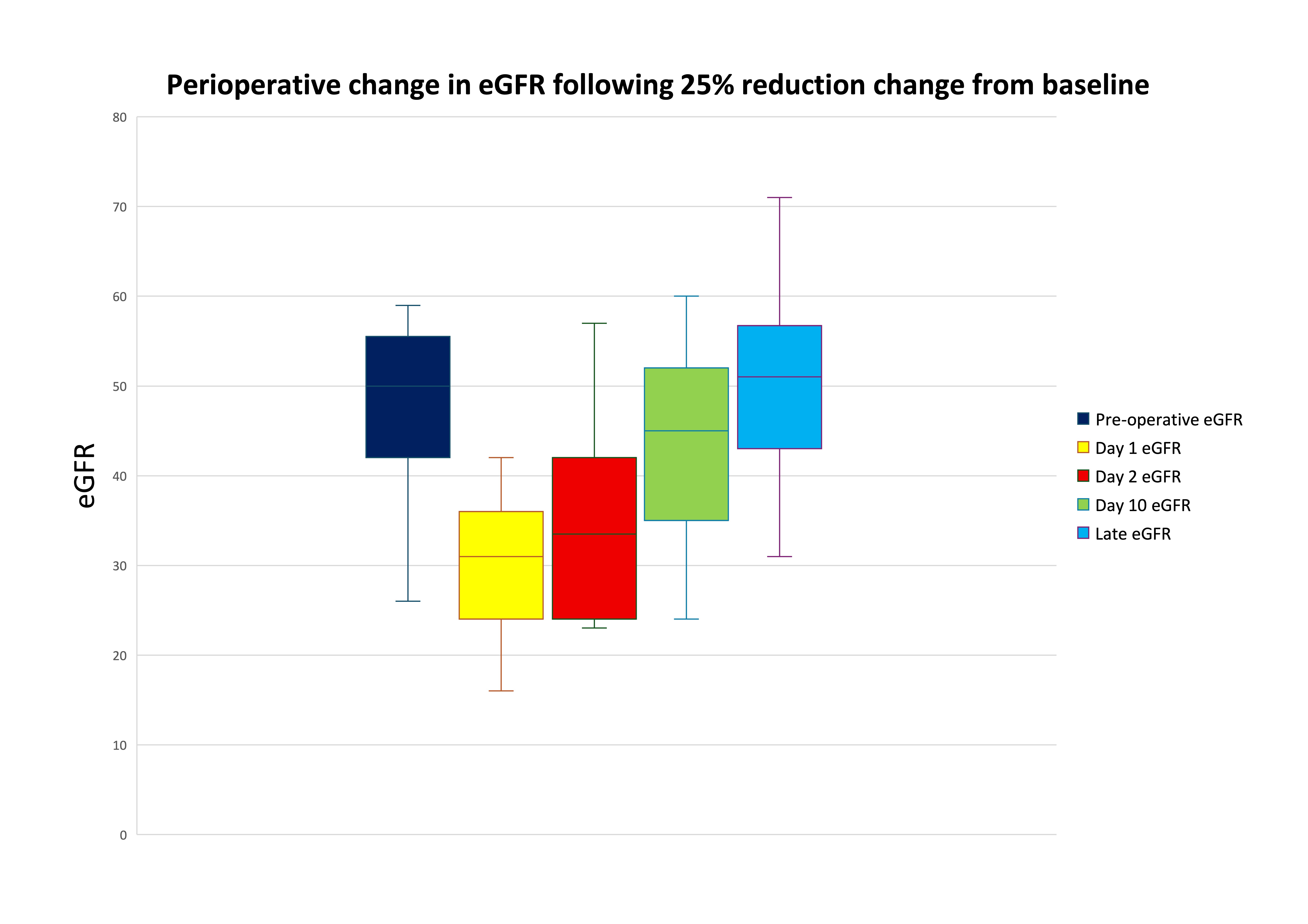

Supplement: Supplementary file 3 — Figure S2. Percentage change in median post‐operative eGFR as compared to baseline eGFR. [file ANS-95-1553-s002.jpg]

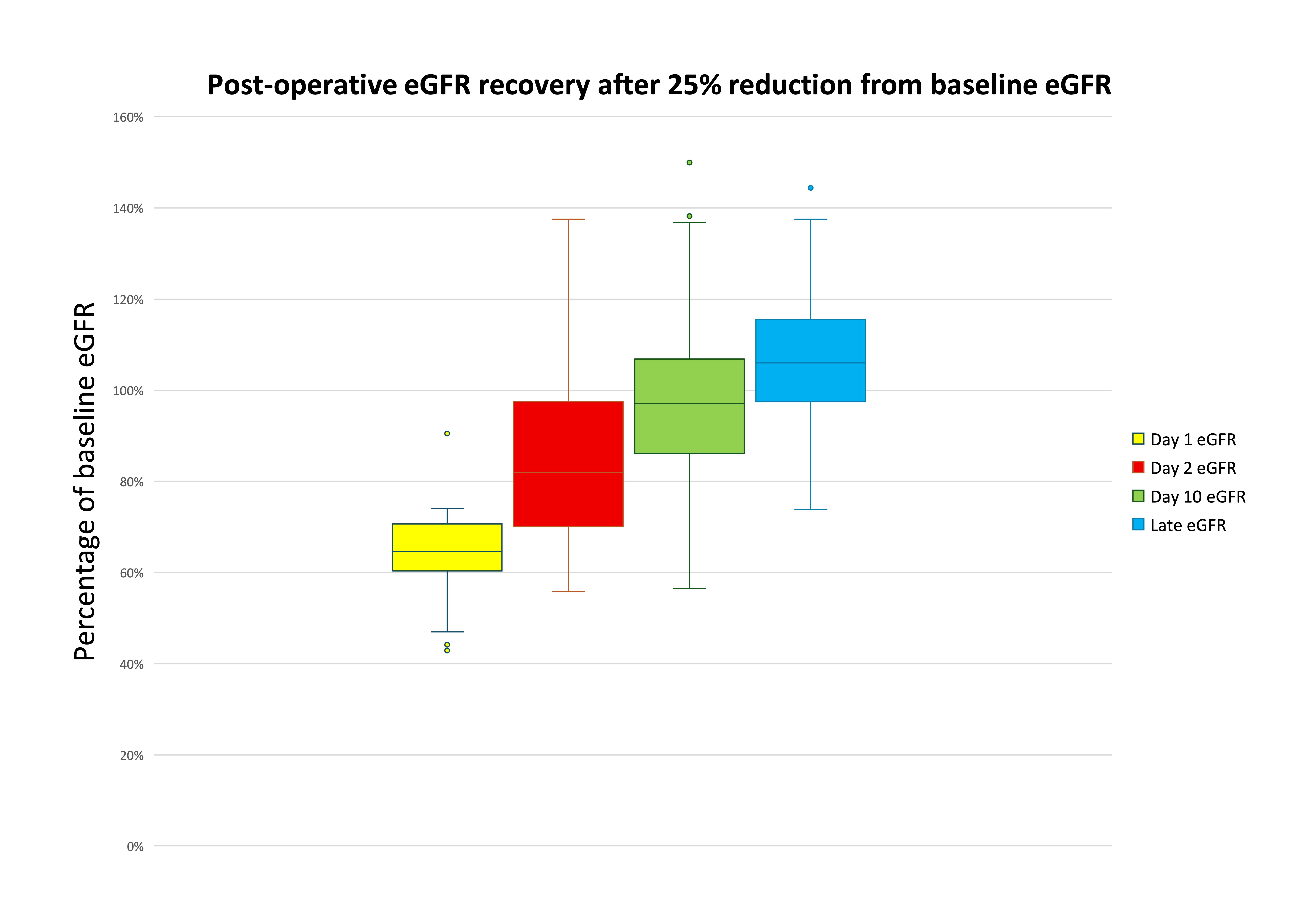

Supplement: Supplementary file 4 — Figure S3. Percentage change in median eGFR following 25% reduction from baseline eGFR. [file ANS-95-1553-s005.jpg]
