# Supplementary material for: Reduced Dose Perioperative Non‐Steroidal Anti‐Inflammatory Drugs in Arthroplasty Patients With Renal Impairment: A Five‐Year Cohort Study
Source: ANZ J Surg. 2025 Jul 25;95(7-8):1553–9. doi: 10.1111/ans.70261 (PMC12413587; doi:10.1111/ans.70261)
Supplement: Supplementary file 5 — Table S1. Cohort demographics. [file ANS-95-1553-s003.docx]

**Table S1 –** Cohort demographics

| **Total Patients (*n*)** | 221 |
| --- | --- |
| **Gender (%)** | |
| Female | 129 (58) |
| Male | 92 (42) |
| **Age at surgery (years)** | |
| Mean (SD) | 78 (8) |
| <60 (%) | 1 (0.45) |
| 60-69 (%) | 38 (17) |
| 70-79 (%) | 85 (38) |
| 80-89 (%) | 85 (38) |
| ≥90 (%) | 12 (5) |
| **Baseline eGFR (mL/min/1.73m^2^)** | |
| Median (range) | 50 (13-59) |
| 45-59 (%) | 153 (69) |
| 30-44 (%) | 56 (25) |
| 15-29 (%) | 11 (5) |
| <15 (%) | 1 (0.45) |
| **Weight (kilograms)** | |
| Mean (SD) | 84 (17) |
| **Length of stay (days)** | |
| Median (range) | 2 (1-54) |
| **Joint replaced (%)** | |
| Knee | 126 (57) |
| Hip | 87 (39) |
| Shoulder | 8 (4) |
| **Surgery volume (%)** | |
| Primary arthroplasty | 201 (91) |
| Revision arthroplasty | 20 (9) |
